# Insulin resistance assessed by estimated glucose disposal rate is associated with all-cause and cardiovascular mortality among postmenopausal women

**DOI:** 10.3389/fendo.2025.1583991

**Published:** 2025-07-30

**Authors:** Han Qian, Dayang Chai, Shouming Zhao

**Affiliations:** Department of Cardiology, The First People’s Hospital of Taicang, Taicang Affiliated Hospital of Soochow University, Taicang, Jiangsu, China

**Keywords:** estimated glucose disposal rate, insulin resistance, all-cause mortality, cardiovascular mortality, postmenopausal women

## Abstract

**Purpose:**

The estimated glucose disposal rate (eGDR) is a noninvasive and practical marker for assessing insulin resistance, but its association with mortality in postmenopausal women remains uncertain.

**Methods:**

A cohort of 9371 postmenopausal women from the National Health and Nutrition Examination Survey (1999-2018) was studied. Baseline eGDR was calculated, and mortality outcomes (all-cause and cardiovascular) were linked to National Death Index (NDI) records up to December 31, 2019. Multivariate Cox regression, restricted cubic splines, and subgroup analyses were employed to assess the relationships between eGDR and mortality.

**Results:**

During a median follow-up of 98 months, 2151 deaths from all causes and 679 from cardiovascular causes were documented. In the multivariable-adjusted Cox model, higher eGDR quartiles were associated with progressively lower all-cause and cardiovascular mortality. In comparison to the lowest eGDR quartile, the highest quartile showed adjusted hazard ratios of 0.765 (0.646-0.906) for all-cause mortality and 0.677 (0.498-0.921) for cardiovascular mortality. A U-shaped relationship between eGDR and all-cause mortality was identified, with an inflection point at 5.11 mg/kg/min. Subgroup analyses revealed a stronger association between eGDR and all-cause as well as cardiovascular mortality in individuals younger than 60 years.

**Conclusions:**

Among postmenopausal women, decreased eGDR, signifying higher insulin resistance, correlates with greater risks of all-cause and cardiovascular mortality.

## 1 Introduction

Menopause, as an inevitable process of aging in women, refers to the cessation of the menstrual cycle caused by anovulation, typically occurring around the age of 50 (1, 2). With increasing life expectancy, it is estimated that women in the United States will spend about one-third of their lives post menopause (3, 4). The decline in estrogen levels following menopause leads to significant physiological changes (5). Epidemiological studies have consistently shown that postmenopausal women are at a higher cardiometabolic risk (6–8). Insulin resistance (IR) is a clinical state of decreased insulin sensitivity and responsiveness (9). Aa a hallmark of metabolic dysfunction, it is a key contributor to the development of chronic diseases such as type 2 diabetes mellitus (T2DM), cardiovascular diseases (CVDs), and certain cancers (9). Among postmenopausal women, the burden of IR is particularly concerning due to the interplay of hormonal changes, aging, and lifestyle factors, which exacerbate metabolic dysfunction and heighten the risk of adverse health outcomes (10, 11). Thus, early interventions targeting IR in postmenopausal women might be essential for mitigating the long-term risks of death (10, 12).

The hyperinsulinemic-euglycemic clamp is widely regarded as the gold standard for assessing IR, but its application in large-scale epidemiological research is limited by its procedural complexity and time demands (13). Additionally, the predictive accuracy of the traditional homeostasis model assessment for insulin resistance (HOMA-IR) might be affected in patients receiving insulin therapy or those with impaired beta-cell function (14). To overcome these shortcomings, researchers have shifted their focus to non-insulin-based indicators for IR assessment (15). The estimated glucose disposal rate (eGDR), calculated using glycated hemoglobin (HbA1c), waist circumference (WC), and hypertension status, has recently emerged as a simple and reliable surrogate marker for IR (16). Previous studies have linked elevated eGDR levels to a lower risk of cardiovascular and cerebrovascular events, along with reduced cardiovascular and all-cause mortality (17–20). However, current research on the prognostic role of the eGDR in postmenopausal women is highly limited. Its potential as a substitute biomarker in healthcare for this group has yet to be determined.

Thus, this study aims to address these gaps in knowledge by investigating the association between eGDR and mortality outcomes in a nationally representative cohort of postmenopausal women.

## 2 Materials and methods

### 2.1 Study population

Data for this analysis were obtained from the National Health and Nutrition Examination Survey (NHANES), conducted by the National Center for Health Statistics at the Centers for Disease Control and Prevention. The survey utilized a stratified, multi-stage random sampling design to ensure a nationally representative sample. Participants took part in physical assessments, completed health and nutrition questionnaires, and underwent laboratory testing. The NHANES protocol was approved by the Ethics Review Board of the National Center for Health Statistics, with all participants providing their written consent.

**Figure 1:**
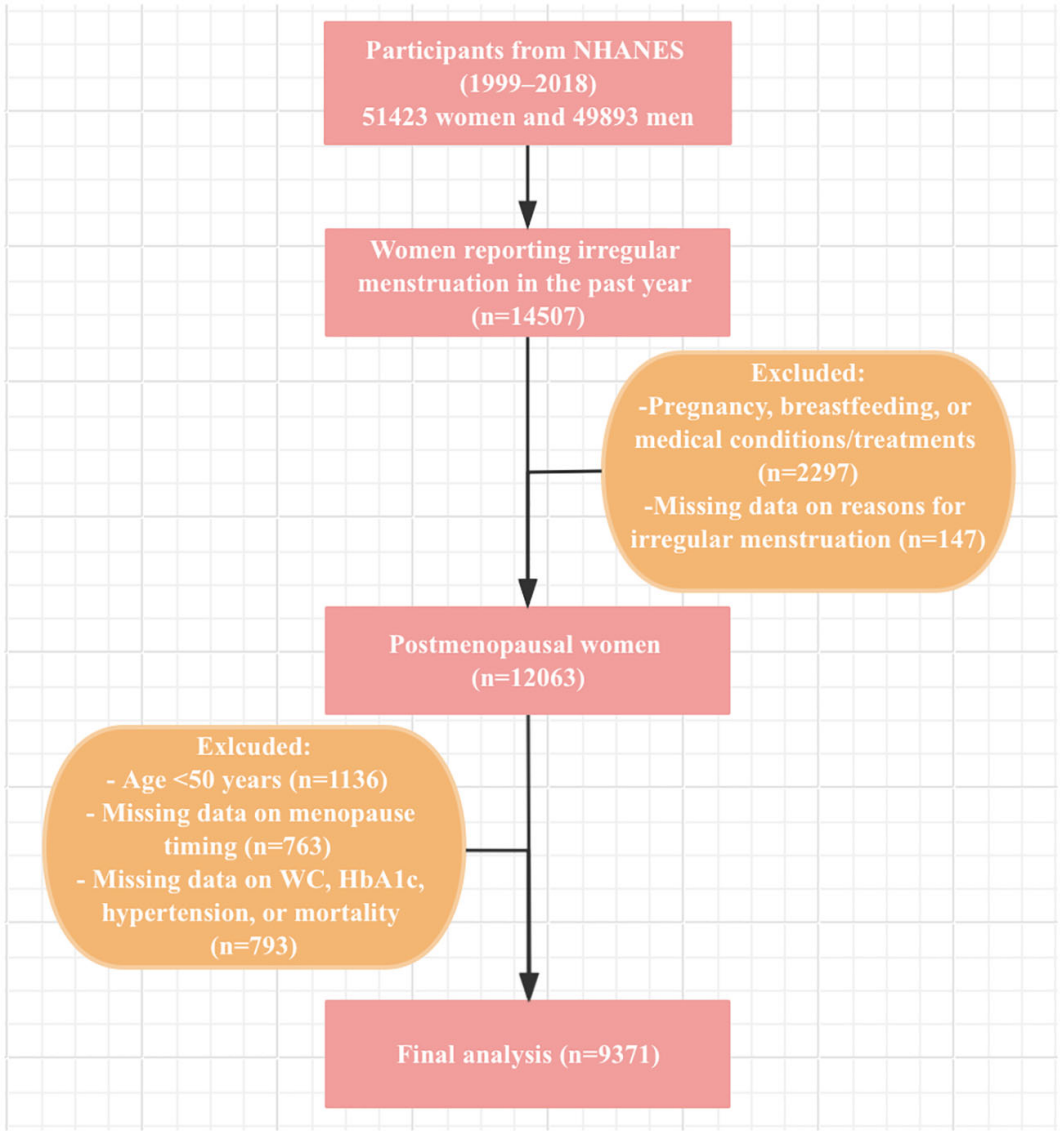
Flow chart of study participants.

Data from 10 NHANES cycles (1999–2000 to 2017-2018) were included in this study. From the 51423 women participants during this period, exclusions were made for women under 50 years old, non-premenopausal women identified via the reproductive health questionnaire, and those with missing data on eGDR calculation, menopause timing, or mortality status. Finally, a total of 9371 postmenopausal women were enrolled in this study. The participant selection process is described in **Figure 1**.

### 2.2 Calculation of eGDR 

The eGDR (mg/kg/min) is calculated using the formula: 21.158 - (0.09 × WC [cm]) − (3.407 × hypertension [1 for yes, 0 for no]) - (0.551 × HbA1c [%]) (21). Participants were categorized into four quartiles based on their eGDR values: Q1 (<4.904), Q2 (4.904-6.352), Q3 (6.352-8.706), and Q4 (>8.706), with Q1 serving as the reference group.

### 2.3 Assessment of menopausal status

Menopausal status was determined based on the responses to the questionnaire on reproductive health (22–24). Participants were first asked: “Had regular periods in past 12 months?” Those who answered “No” were further asked about the reasons for irregular menstruation. For participants who responded “No,” the reasons were further clarified to include acknowledgment of either undergoing a hysterectomy or menopause/change of life. Participants experiencing irregular menstruation due to pregnancy, breastfeeding, or medical conditions/treatments were excluded from the analysis. The age at menopause was determined by asking participants the question: “Age at last menstrual period”. We also excluded women under the age of 50.

### 2.4 Assessment of mortality

Mortality status was tracked through December 31, 2019, using the NHANES Public-Use Linked Mortality File, which applies a probabilistic algorithm to match participants with the National Death Index (NDI). Follow-up time was defined as the interval between the participant’s baseline examination and the last date they were confirmed alive or censored in the mortality file. All-cause mortality was considered, while disease-specific deaths were identified using the International Statistical Classification of Diseases, 10th Revision (ICD-10). Cardiovascular mortality was defined as deaths caused by major cardiovascular and cerebrovascular conditions, identified by ICD-10 codes I00–I09, I11, I13, I20–I51, and I60–I69.

### 2.5 Assessment of covariates

The study analyzed covariates such as demographic characteristics, physical examination results, laboratory blood tests, and medical history. (1) Demographic data included age, age at menopause, race, marital status, education level, family income-to-poverty ratio (PIR), smoking status, and alcohol consumption. Race classifications were Mexican American, non-Hispanic black, non-Hispanic white, other Hispanic, and other. Education levels were classed as less than high school, high school, and some college or above. PIR was divided into groups of <1.3, 1.3-3.5, and ≥3.5. Smoking status was categorized into never, former, or current smoker. Alcohol consumption was identified as having at least 12 alcoholic drinks per year. (2) Physical examination measurements included body mass index (BMI, kg/m^2^) and WC (cm). BMI, calculated as weight in kilograms divided by height in meters squared, was classified as <25, 25-30, and ≥30kg/m^2^. (3) Laboratory indicators included fasting plasma glucose (FPG, mg/dL), hemoglobin A1c (HbA1c, %), albumin (g/L), aspartate aminotransferase (ALT, U/L), alanine aminotransferase (AST, U/L), triglyceride (TG, mg/dL), total cholesterol (TC, mg/dL), high-density lipoprotein cholesterol (HDL-c, mg/dL), low-density lipoprotein cholesterol (LDL-c, mg/dL), serum uric acid (SUA, mg/dL), serum creatinine (SCr, μmol/L), and the estimated glomerular filtration rate (eGFR, ml/min/1.73m²). The eGFR was calculated using the Chronic Kidney Disease Epidemiology Collaboration (CKD-EPI) equation, which incorporates age, gender, race, and SCr levels (25). (4) Medical history covered diabetes and hypertension. Diabetes was defined based on self-reported diagnosis, the use of insulin or oral hypoglycemic agents, FPG levels ≥126mg/dL, or HbA1c levels ≥6.5% (26). Hypertension was defined as a self-reported diagnosis, three average systolic blood pressure (SBP) ≥140mmHg, three average diastolic blood pressure (DBP) ≥90mmHg, or use of antihypertensive medications.

### 2.6 Statistical analysis

Our analyses adhered to the NHANES data analysis standards by integrating sample weights, clustering, and stratification to address the intricate sampling design. Continuous variables were reported as mean and standard deviation (SD), while categorical variables were presented as unweighted counts and weighted percentages. We assume that the data are missing at random and applied the random forest method for iterative imputation of the missing covariates. Continuous variables were compared using the Mann-Whitney test, and categorical variables were analyzed with the Rao-Scott chi-square test. Cox proportional hazards regression models were applied to calculate hazard ratios (HRs) and 95% confidence intervals (CIs) for the association between eGDR and all-cause or cardiovascular mortality in postmenopausal women. Four models were developed: model 1 was unadjusted, model 2 adjusted for age and race, model 3 further adjusted for marital status, education level, PIR, smoking history, and alcohol consumption, and model 4 additionally adjusted for age at menopause, BMI, diabetes, albumin, AST, ALT, TG, HDL-c, LDL-c, SUA, and eGFR. Collinearity among covariates in model was not significant, as evidenced by variance inflation factors (VIFs), all of which were below 3 (**Supplementary Materials: Supplementary Table S1**). The Kaplan-Meier method was applied to estimate survival curves, and survival differences in all-cause and cardiovascular mortality across eGDR quantiles were analyzed using the log-rank test. Cox proportional hazards regression models with restricted cubic splines (RCS) were used to identify the nonlinear relationship between eGDR and mortality risk. For nonlinear relationships, the threshold is determined by testing all values and selecting the one with the highest likelihood. A two-piecewise Cox model is used on both sides of the inflection point to analyze the link between eGDR and mortality risk. Stratified analyses were conducted for significant covariates, considering potential effect modifiers like age, race, marital status, education level, PIR, smoking history, alcohol consumption, BMI, diabetes, and eGFR. To evaluate the incremental predictive value of incorporating eGDR into the basic model, the C-statistic, net reclassification improvement (NRI), and integrated discrimination improvement (IDI) were applied. Statistical analysis was conducted with R software (version 4.2.0), and a two-tailed ***P*** value of 0.05 was used to define significance.

## 3 Results

### 3.1 Baseline characteristics of study population

A total of 9371 postmenopausal women were included in the study (Mean [SD] age: 65.82 [9.32] years). Of these, 1412 (weighted 4.32%) were Mexican American, 1780 (weighted 9.22%) were non-Hispanic Black, 4643 (weighted 76.89%) were non-Hispanic White, 874 (weighted 4.07%) were other Hispanic, and 662 (weighted 5.50%) were categorized as other. Demographic characteristics were stratified by survival status (**Table 1**). Non-survivors were older, had a younger age at menopause, were more likely to be Non-Hispanic White, unmarried, less educated, had a lower PIR, smoked, consumed less alcohol, and had lower BMI, WC, ALT, albumin, LDL-c, and eGFR levels (***P*** <0.01). They also showed higher FPG, AST, SUA, and SCr levels, along with a history of diabetes and hypertension (***P*** <0.001). Baseline eGDR levels were significantly lower in non-survivors compared to survivors (***P*** <0.001).

**Table 1:** Baseline characteristics of participants stratified by survival status.

Characteristic	Overall (n=9371)	Survivors (n=7220)	Non-survivors (n=2151)	*P* value
Age (years)	65.82 (9.32)	63.76 (8.37)	72.72 (9.02)	<0.001
Age at menopause (years)	45.94 (7.90)	46.07 (7.75)	45.52 (8.38)	0.004
Race, n%				<0.001
Mexican American	1412 (4.32%)	1139 (4.53%)	273 (3.17%)	
Non-Hispanic Black	1780 (9.22%)	1437 (9.31%)	343 (8.75%)	
Non-Hispanic White	4643 (76.89%)	3278 (75.97%)	1365 (82.00%)	
Other Hispanic	874 (4.07%)	770 (4.40%)	104 (2.28%)	
Other Races	662 (5.50%)	596 (5.80%)	66 (3.80%)	
Married/Living with partner, n%				<0.001
No	4664 (41.73%)	3319 (38.64%)	1345 (58.82%)	
Yes	4707 (58.27%)	3901 (61.36%)	806 (41.18%)	
Education level, n%				<0.001
<High school	2722 (16.56%)	1909 (14.17%)	813 (29.79%)	
High school	2389 (27.25%)	1804 (27.01%)	585 (28.58%)	
Some college or above	4260 (56.19%)	3507 (58.82%)	753 (41.63%)	
PIR, n%				<0.001
<1.3	2482 (16.80%)	1787 (14.88%)	695 (27.45%)	
≥1.3, <3.5	4212 (40.68%)	3159 (39.28%)	1053 (48.46%)	
≥ 3.5	2677 (42.51%)	2274 (45.84%)	403 (24.10%)	
Smoking history, n%				<0.001
Never	5664 (57.89%)	4515 (59.27%)	1149 (50.31%)	
Current	1255 (13.56%)	930 (13.07%)	325 (16.27%)	
Former	2452 (28.55%)	1775 (27.66%)	677 (33.42%)	
Alcohol consumption, n%				<0.001
No	4115 (34.58%)	3023 (32.48%)	1052 (46.19%)	
Yes	5256 (65.42%)	4197 (67.52%)	1059 (53.81%)	
BMI (kg/m^2^), n%				<0.001
<25	2439 (28.62%)	1759 (27.52%)	680 (34.70%)	
≥25, <30	2973 (31.65%)	2279 (31.79%)	694 (30.86%)	
≥30	3959 (39.74%)	3182 (40.70%)	777 (34.43%)	
WC (cm)	99.44 (15.03)	99.83 (14.99)	98.15 (15.10)	<0.001
FPG (mg/dL)	114.21 (34.06)	112.89 (32.06)	118.63 (39.74)	<0.001
HbA1c (%)	6.00 (1.13)	5.99 (1.10)	6.03 (1.23)	0.105
Albumin (g/L)	41.56 (3.09)	41.69 (3.01)	41.13 (3.31)	<0.001
ALT (U/L)	21.90 (12.65)	22.32 (12.68)	20.51 (12.46)	<0.001
AST (U/L)	24.65 (11.54)	24.40 (10.63)	25.48 (14.15)	<0.001
TG (mg/dL)	152.19 (96.51)	151.16 (94.72)	155.66 (102.21)	0.057
TC (mg/dL)	208.01 (41.81)	208.02 (41.13)	208.00 (44.02)	0.984
HDL (mg/dL)	59.10 (16.80)	59.15 (16.34)	58.95 (18.25)	0.636
LDL (mg/dL)	121.60 (32.09)	122.25 (32.05)	119.42 (32.12)	<0.001
SUA (mg/dL)	5.24 (1.38)	5.16 (1.31)	5.50 (1.58)	<0.001
SCr (μmol/L)	74.45 (33.49)	71.38 (22.11)	84.76 (55.74)	<0.001
eGFR (ml/min/1.73m²)	79.47 (20.40)	82.30 (18.73)	69.95 (22.76)	<0.001
eGDR (mg/kg/min)	6.68 (2.52)	6.74 (2.55)	6.45 (2.41)	<0.001
Diabetes, n%				<0.001
No	7058 (80.51%)	5557 (82.30%)	1501 (70.61%)	
Yes	2313 (19.49%)	1663 (17.70%)	650 (29.39%)	
Hypertension, n%				<0.001
No	3244 (39.91%)	2704 (42.46%)	540 (25.78%)	
Yes	6127 (60.09%)	4516 (57.54%)	1611 (74.22%)	

Data are presented as mean (SD) or counts (weighted percentages).

PIR, family income-to-poverty ratio; BMI, body mass index; WC, waist circumference; FPG, fasting plasma glucose; HbA1c, hemoglobin A1c; ALT, aspartate aminotransferase; AST, alanine aminotransferase; TG, triglyceride; TC, total cholesterol; HDL-c, high-density lipoprotein cholesterol; LDL-c, low-density lipoprotein cholesterol; SUA, serum uric acid; SCr, serum creatinine; eGFR, estimated glomerular filtration rate; eGDR, estimated glucose disposal rate.

On the other hand, **Table 2** presents the baseline characteristics by eGDR quartiles. Participants in higher eGDR quartiles were younger, had an older age at menopause, were more likely to be non-Hispanic White, married, better educated, had a higher PIR, were nonsmokers, consumed alcohol, and had lower BMI and WC compared to those in the lowest quartile (***P*** <0.001). Biochemical indicators also varied significantly across groups. Those in the highest quartile exhibited lower levels of FPG, HbA1c, ALT, TG, SUA, and SCr, a lower prevalence of diabetes and hypertension, and higher levels of albumin, TC, HDL-c, LDL-c, eGFR, and eGDR compared to the first quartile (***P*** <0.001). Significant differences in all-cause and cardiovascular mortality were also noted among eGDR quartiles (***P*** <0.001).

**Table 2:** Baseline characteristics according to eGDR quartiles (Q1-Q4).

Characteristic	Q1 (n=2343)	Q2 (n=2342)	Q3 (n=2343)	Q4 (n=2343)	*P* value
Age (years)	65.50 (8.42)	67.84 (9.28)	66.83 (9.65)	63.10 (9.20)	<0.001
Age at menopause (years)	45.19 (8.41)	45.92 (7.92)	46.07 (7.86)	46.58 (7.31)	<0.001
Race, n%					<0.001
Mexican American	372 (5.28%)	365 (4.61%)	359 (4.25%)	316 (3.44%)	
Non-Hispanic Black	700 (16.16%)	467 (10.15%)	358 (7.67%)	255 (4.64%)	
Non-Hispanic White	979 (70.72%)	1148 (75.95%)	1228 (78.09%)	1288 (81.20%)	
Other Hispanic	202 (3.62%)	220 (4.36%)	193 (3.38%)	259 (4.75%)	
Other Races	90 (4.22%)	142 (4.93%)	205 (6.60%)	225 (5.96%)	
Married/Living with partner, n%					<0.001
No	1297 (47.57%)	1218 (45.69%)	1161 (41.13%)	988 (34.87%)	
Yes	1046 (52.43%)	1124 (54.31%)	1182 (58.87%)	1355 (65.13%)	
Education level, n%					<0.001
<High school	798 (20.92%)	762 (19.77%)	636 (15.61%)	526 (11.67%)	
High school	611 (30.06%)	627 (29.13%)	616 (27.59%)	535 (23.45%)	
Some college or above	934 (49.01%)	953 (51.10%)	1091 (56.80%)	1282 (64.88%)	
PIR, n%					<0.001
<1.3	782 (23.20%)	673 (19.15%)	584 (15.53%)	443 (11.33%)	
≥1.3, <3.5	1087 (45.17%)	1098 (43.39%)	1080 (44.57%)	947 (32.09%)	
≥ 3.5	474 (31.64%)	571 (37.46%)	679 (39.90%)	953 (56.58%)	
Smoking history, n%					<0.001
Never	1363 (56.21%)	1475 (59.88%)	1387 (55.68%)	1439 (59.48%)	
Current	288 (11.69%)	271 (11.18%)	358 (16.80%)	338 (14.07%)	
Former	692 (32.10%)	596 (28.94%)	598 (27.53%)	566 (26.44%)	
Alcohol consumption, n%					<0.001
No	1131 (41.22%)	1085 (37.45%)	984 (32.53%)	915 (29.15%)	
Yes	1212 (58.78%)	1257 (62.55%)	1359 (67.47%)	1428 (70.85%)	
BMI (kg/m^2^), n%					<0.001
<25	25 (0.64%)	225 (9.05%)	1069 (45.45%)	1120 (50.33%)	
≥25, <30	256 (9.12%)	1168 (51.81%)	611 (24.60%)	938 (39.01%)	
≥30	2062 (90.23%)	949 (39.14%)	663 (29.95%)	285 (10.65%)	
WC (cm)	116.10 (11.51)	100.08 (7.46)	93.07 (13.83)	88.51 (9.27)	<0.001
FPG (mg/dL)	138.57 (49.80)	112.03 (26.02)	106.02 (21.65)	100.21 (12.39)	<0.001
HbA1c (%)	6.80 (1.63)	5.92 (0.90)	5.73 (0.75)	5.55 (0.43)	<0.001
Albumin (g/L)	40.46 (3.08)	41.71 (2.90)	41.81 (3.15)	42.26 (2.92)	<0.001
ALT (U/L)	23.06 (14.13)	21.66 (12.50)	21.50 (11.97)	21.38 (11.81)	<0.001
AST (U/L)	24.55 (12.81)	24.72 (13.31)	24.80 (10.02)	24.51 (9.55)	0.801
TG (mg/dL)	175.42 (112.57)	161.61 (105.80)	140.56 (77.91)	131.18 (78.27)	<0.001
TC (mg/dL)	199.36 (42.87)	207.62 (42.20)	210.26 (41.16)	214.80 (39.46)	<0.001
HDL (mg/dL)	52.48 (13.34)	57.60 (15.49)	62.40 (18.20)	63.93 (17.31)	<0.001
LDL (mg/dL)	115.59 (31.37)	121.47 (32.30)	122.35 (31.88)	127.01 (31.78)	<0.001
SUA (mg/dL)	5.88 (1.47)	5.41 (1.33)	5.04 (1.30)	4.61 (1.08)	<0.001
SCr (μmol/L)	79.75 (41.87)	76.48 (31.03)	74.33 (38.15)	67.26 (15.19)	<0.001
eGFR (ml/min/1.73m²)	76.94 (22.06)	76.50 (20.71)	79.17 (20.41)	85.25 (16.87)	<0.001
eGDR (mg/kg/min)	3.60 (1.13)	5.64 (0.41)	7.36 (0.68)	10.10 (0.86)	<0.001
Diabetes, n%					<0.001
No	1045 (50.60%)	1790 (79.70%)	2020 (89.47%)	2203 (96.01%)	
Yes	1298 (49.40%)	552 (20.30%)	323 (10.53%)	140 (3.99%)	
Hypertension, n%					<0.001
No	33 (1.35%)	106 (4.45%)	784 (35.16%)	2321 (99.32%)	
Yes	2310 (98.65%)	2236 (95.55%)	1559 (64.84%)	22 (0.68%)	
All-cause mortality, n%	560 (17.31%)	587 (18.63%)	588 (16.43%)	416 (10.38%)	<0.001
Cardiovascular mortality, n%	189 (5.71%)	202 (6.17%)	176 (4.74%)	112 (2.63%)	<0.001

Data are presented as mean (SD) or counts (weighted percentages).

PIR, family income-to-poverty ratio; BMI, body mass index; WC, waist circumference; FPG, fasting plasma glucose; HbA1c, hemoglobin A1c; ALT, aspartate aminotransferase; AST, alanine aminotransferase; TG, triglyceride; TC, total cholesterol; HDL-c, high-density lipoprotein cholesterol; LDL-c, low-density lipoprotein cholesterol; SUA, serum uric acid; SCr, serum creatinine; eGFR, estimated glomerular filtration rate; eGDR, estimated glucose disposal rate.

### 3.2 Survival patterns of postmenopausal women in different eGDR quartiles

**Figure 2:**
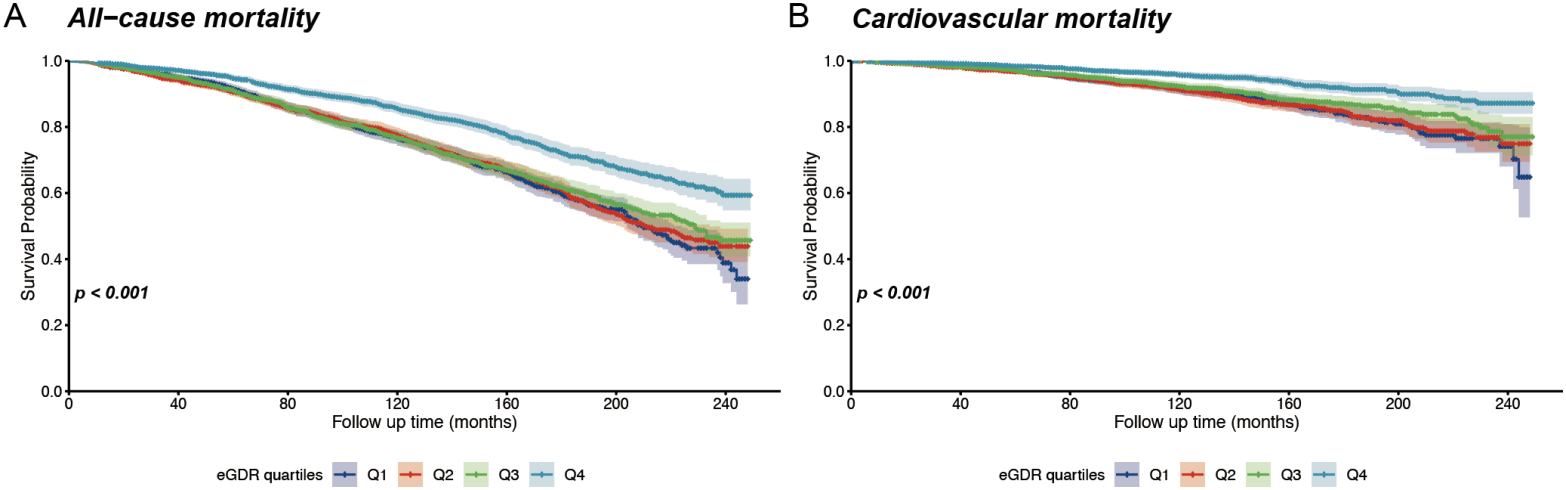
Kaplan-Meier survival curves for all-cause and cardiovascular mortality according to eGDR quantiles. **(A)** Overall survival stratified by eGDR quartiles; **(B)** Cardiovascular-specific survival stratified by eGDR quartiles.

Over a median follow-up of 98 months, 2151 all-cause deaths occurred, including 679 cardiovascular disease-related deaths. In postmenopausal women, Kaplan-Meier survival analysis demonstrated that individuals in the lowest quartile of eGDR exhibited significantly reduced survival probabilities over time compared to those in the highest quartile (**Figure 2**). This pattern was observed for both all-cause mortality and cardiovascular mortality, with a log-rank test P-value <0.001. There is a strong association between lower eGDR and elevated mortality risk in this population.

### 3.3 Association of eGDR with mortality in postmenopausal women

**Table 3** presents the results of four Cox proportional hazards regression models evaluating eGDR levels and mortality risk. Models 1, 2, and 3 indicate a significant downward trend in the relationship between eGDR and all-cause as well as cardiovascular mortality (***P*** for trend < 0.001). In Model 4, after adjusting for age, race, marital status, education level, PIR, smoking history, alcohol consumption, age at menopause, BMI, diabetes, albumin, AST, ALT, TG, HDL-c, LDL-c, SUA, and eGFR, the HRs and 95% CIs for all-cause mortality across Q1, Q2, Q3, and Q4 groups were 1.000 (reference), 0.839 (0.733-0.961), 0.872 (0.748-1.018), and 0.765 (0.646-0.906), respectively, with a trend test P-value of 0.008. For cardiovascular mortality, the HRs (95% CIs) for Q1, Q2, Q3, and Q4 groups were 1.000 (reference), 0.865 (0.686-1.092), 0.782 (0.593-1.032), and 0.677 (0.498-0.921), with a trend test P-value of 0.012.

**Table 3:** Associations between eGDR and risk of all-cause and cardiovascular mortality in postmenopausal women.

eGDR quantile	Q1 (<4.904)	Q2 (4.904-6.352)		Q3 (6.352-8.706)		Q4 (>8.706)		*P* for trend
Mortality outcome	HR (95% CI)	HR (95% CI)	*P* value	HR (95% CI)	*P* value	HR (95% CI)	*P* value	
All-cause mortality								
No. of cases/N	560/2343	587/2342		588/2343		416/2343		
Model 1	Ref.	0.983 (0.875, 1.103)	0.768	0.947 (0.844, 1.064)	0.360	0.599 (0.527, 0.680)	<0.001	<0.001
Model 2	Ref.	0.749 (0.666, 0.842)	<0.001	0.794 (0.706, 0.892)	<0.001	0.658 (0.579, 0.749)	<0.001	<0.001
Model 3	Ref.	0.758 (0.674, 0.853)	<0.001	0.801 (0.711, 0.901)	<0.001	0.679 (0.596, 0.773)	<0.001	<0.001
Model 4	Ref.	0.839 (0.733, 0.961)	0.011	0.872 (0.748, 1.018)	0.082	0.765 (0.646, 0.906)	0.002	0.008
Cardiovascular mortality								
No. of cases/N	189/2343	202/2342		176/2343		112/2343		
Model 1	Ref.	1.001 (0.821, 1.220)	0.994	0.840 (0.684, 1.031)	0.096	0.477 (0.378, 0.603)	<0.001	<0.001
Model 2	Ref.	0.706 (0.577, 0.863)	<0.001	0.661 (0.536, 0.814)	<0.001	0.522 (0.411, 0.662)	<0.001	<0.001
Model 3	Ref.	0.721 (0.589, 0.882)	0.002	0.679 (0.550, 0.838)	<0.001	0.546 (0.429, 0.694)	<0.001	<0.001
Model 4	Ref.	0.865 (0.686, 1.092)	0.223	0.782 (0.593, 1.032)	0.083	0.677 (0.498, 0.921)	0.013	0.012

Estimates are hazard ratios (95%CI) from Cox proportional hazard models.

Model 1: no adjusted. Model 2: adjusted for age and race. Model 3: adjusted for age, race, marital status, education level, PIR, smoking history, and alcohol consumption. Model 4: adjusted for age, race, marital status, education level, PIR, smoking history, alcohol consumption, age at menopause, BMI, diabetes, albumin, AST, ALT, TG, HDL-c, LDL-c, SUA, and eGFR.

HR, hazard ratio; 95%CI, 95% confidence interval.

### 3.4 Non-linear trend between eGDR and mortality in postmenopausal women

**Figure 3:**
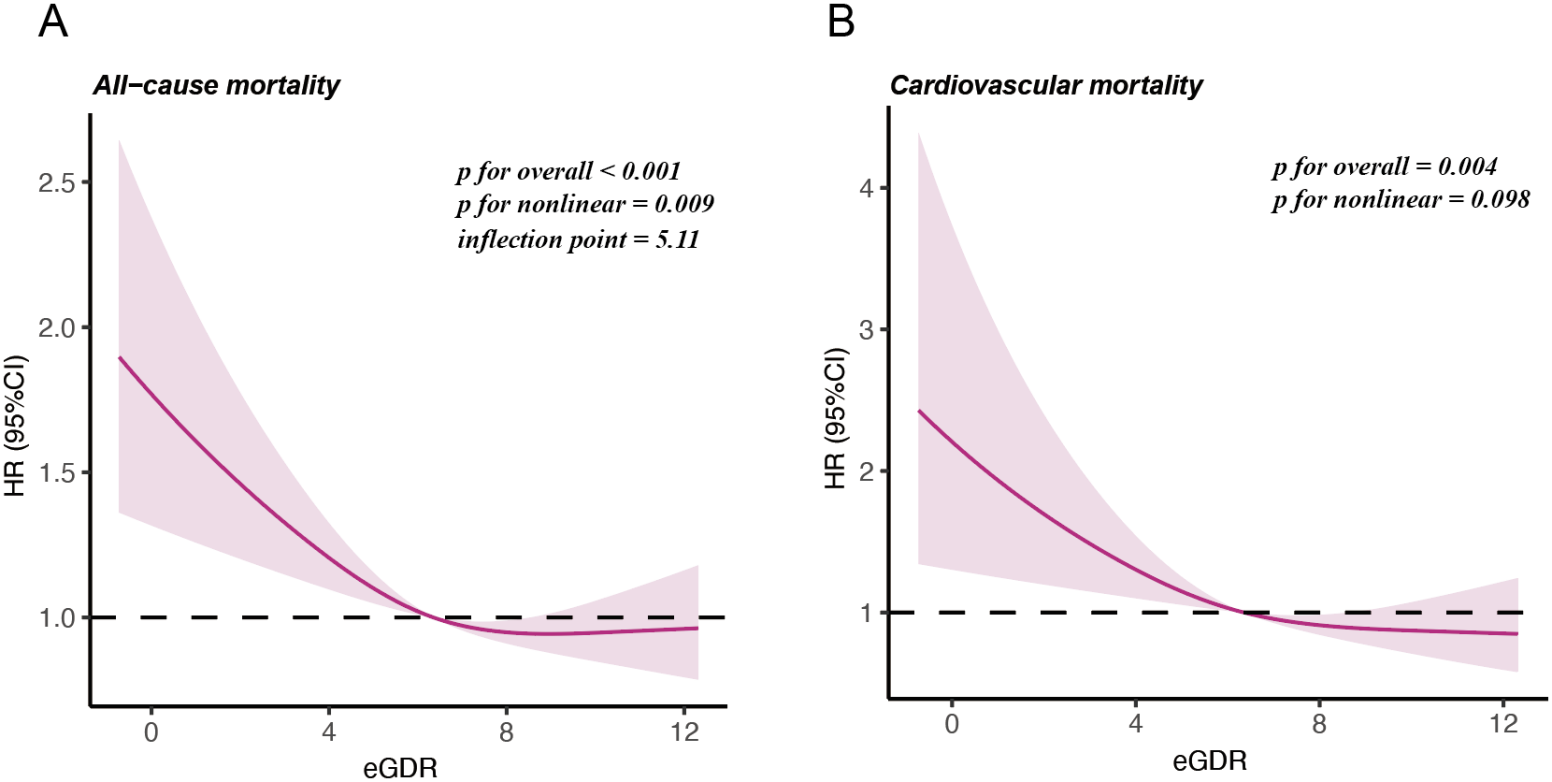
Restricted cubic splines illustrating the relationship between eGDR and mortality outcomes. **(A)** Nonlinear relationship between eGDR and all-cause mortality. **(B)** Linear relationship between eGDR and cardiovascular mortality.

Cox proportional hazards regression models with RCS were applied to assess the non-linear relationship between eGDR and mortality in postmenopausal women (**Figure 3**). After adjusting for age, race, marital status, education level, PIR, smoking history, alcohol consumption, age at menopause, BMI, diabetes, albumin, AST, ALT, TG, HDL-c, LDL-c, SUA, and eGFR, the RCS analysis demonstrated a U-shaped relationship between eGDR and all-cause mortality, with an inflection point identified at eGDR = 5.11mg/kg/min (***P*** for nonlinear = 0.009). Subsequent analyses using segmented regression detailed in **Supplementary Materials: Supplementary Table S2**. The eGDR was linearly associated with cardiovascular mortality among postmenopausal women (***P*** for nonlinear = 0.098).

### 3.5 Stratified analyses

**Figure 4:**
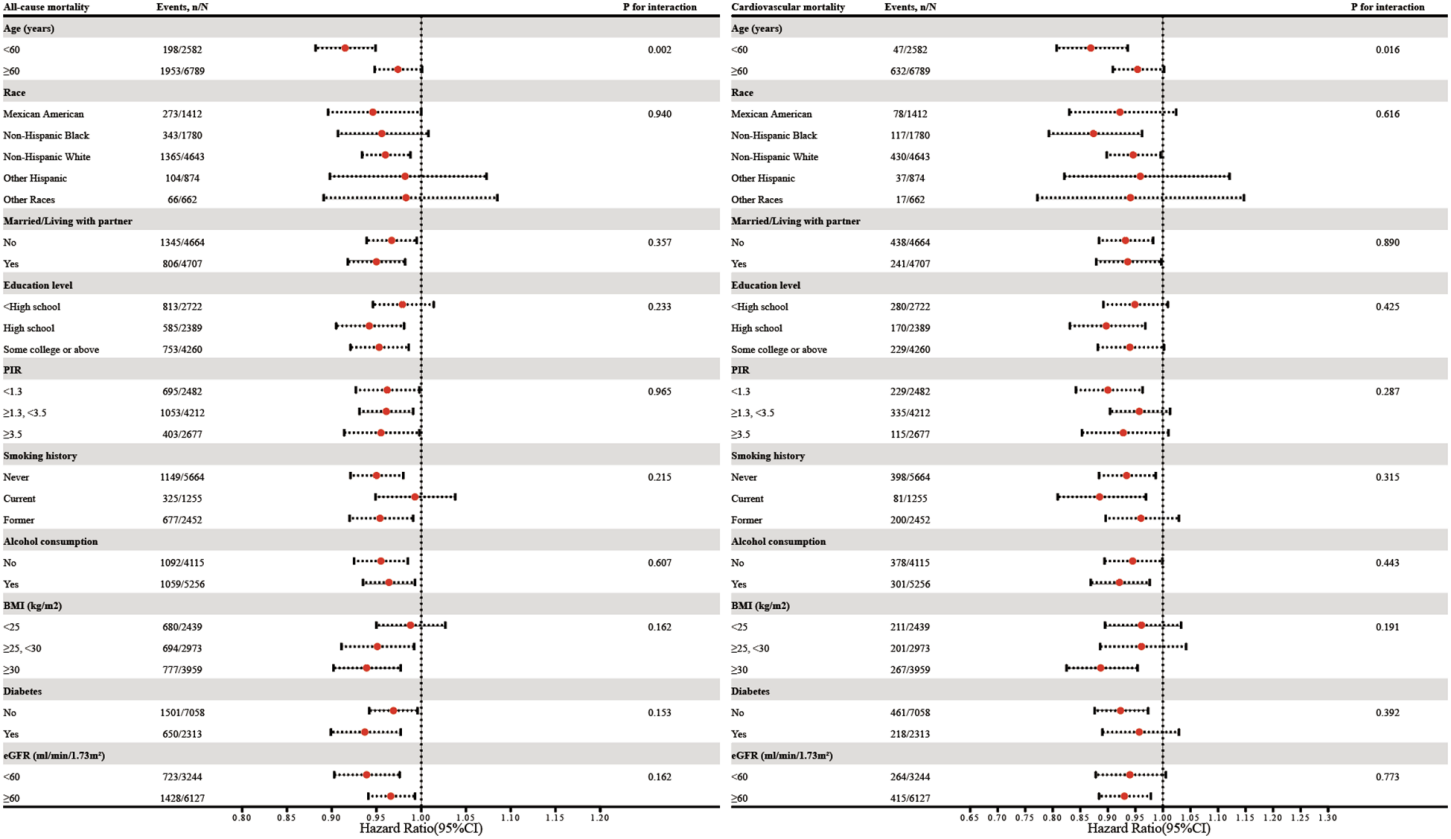
Subgroup analysis of the association between eGDR and all-cause as well as cardiovascular mortality. Adjustments were made for covariates including age, race, marital status, education level, PIR, smoking history, alcohol consumption, age at menopause, BMI, diabetes, albumin, AST, ALT, TG, HDL-c, LDL-c, SUA, and eGFR, except for subgroup factors.

**Figure 4** shows subgroup analyses of the relationship between eGDR (as a continuous variable) and all-cause and cardiovascular mortality, stratified by age, race, marital status, education level, PIR, smoking history, alcohol consumption, BMI, diabetes, and eGFR. Age was found to have a notable leverage on the eGDR-mortality relationship. The interaction analysis indicated that the association of eGDR with all-cause and cardiovascular mortality was stronger in individuals younger than 60 years compared to those aged 60 years or older (***P*** for interaction = 0.002 for all-cause mortality and 0.016 for cardiovascular mortality, respectively).

### 3.6 Incremental predictive value of eGDR

**Table 4** summarizes the incremental predictive values of eGDR and other IR measures, such as the triglyceride-glucose index (TyG) and TG/HDL-c, for all-cause and cardiovascular mortality. The basic model was developed according to age, race, marital status, education level, PIR, smoking history, alcohol consumption, age at menopause, BMI, diabetes, albumin, AST, ALT, LDL-c, SUA, and eGFR. Adding eGDR, TyG, and TG/HDL-c significantly improved the base model’s C-statistics (***P*** <0.05). Additionally, NRI analysis showed significant reclassification improvements for eGDR in predicting both all-cause and cardiovascular mortality (***P*** <0.001), and its inclusion enhanced discriminative power for cardiovascular mortality (***P*** <0.001).

**Table 4:** Model discrimination and risk reclassification for all-cause and cardiovascular mortality.

Analytical model	C-statistic		NRI		IDI	
Mortality outcome	95%CI	*P* value	95%CI	*P* value	95%CI	*P* value
All-cause mortality						
Basic model	0.774 (0.749 - 0.797)		Ref.		Ref.	
Basic model + TG/HDL-c	0.775 (0.750 - 0.798)	0.019	0.070 (0.024 - 0.117)	0.004	0.026 (0.007 - 0.045)	<0.001
Basic model + TyG	0.776 (0.751 - 0.799)	0.012	0.057 (0.012 - 0.106)	0.026	0.014 (-0.010 - 0.037)	0.284
Basic model + eGDR	0.778 (0.754 - 0.802)	<0.001	0.131 (0.086 - 0.177)	<0.001	0.021 (-0.003 - 0.044)	0.088
Cardiovascular mortality						
Basic model	0.768 (0.743 - 0.792)		Ref.		Ref.	
Basic model + TG/HDL-c	0.769 (0.744 - 0.793)	0.003	0.068 (0.024 - 0.114)	0.006	0.030 (0.006 - 0.056)	0.010
Basic model + TyG	0.770 (0.744 - 0.793)	0.020	0.054 (0.005 - 0.102)	0.026	0.034 (-0.007 - 0.075)	0.098
Basic model + eGDR	0.773 (0.746 - 0.795)	<0.001	0.167 (0.122 - 0.214)	<0.001	0.102 (0.053 - 0.150)	<0.001

Adjustments in the basic model were made for factors such as age, race, marital status, education level, PIR, smoking history, alcohol consumption, age at menopause, BMI, diabetes, albumin, AST, ALT, LDL-c, SUA, and eGFR.

95%CI, 95%confidence interval; NRI, net reclassification improvement; IDI, integrated discrimination improvement; TG/HDL-c, triglycerides to high-density lipoprotein cholesterol ratio; TyG, triglyceride-glucose index; eGDR, estimated glucose disposal rate.

### 3.7 Sensitivity analysis

Sensitivity analyses were performed to check the robustness of the results: (1) excluding those who died during the first two years (**Supplementary Materials: Supplementary Table S3**), (2) excluding participants with baseline diabetes (**Supplementary Materials: Supplementary Table S4**), and (3) excluding participants with missing covariates (**Supplementary Materials: Supplementary Table S5**). All analyses produced results consistent with the main findings.

## 4 Discussion

This is the first large-scale retrospective cohort study to assess the relationship between eGDR levels and the risks of all-cause and cardiovascular mortality in postmenopausal women. Higher eGDR levels are independently linked to reduced risks of both outcomes and show strong predictive potential. RCS curves suggest a nonlinear association with all-cause mortality and a linear association with cardiovascular mortality. The eGDR could serve as a surrogate marker for clinical management in postmenopausal women.

IR in postmenopausal women is a precursor to various metabolic disorders (27). In terms of mechanism, estrogen plays a critical role in glucose homeostasis (28). It has been demonstrated that estrogen enhances insulin sensitivity by regulating glucose transporters, such as glucose transporter type 4 (GLUT4), and improving mitochondrial function in skeletal muscle and adipose tissue (28–30). The loss of estrogen during menopause disrupts these processes, leading to IR. Moreover, the increased androgen-to-estrogen ratio in postmenopausal women may exacerbate visceral fat accumulation, which is also a key driver of IR (31). Therefore, an efficient and effective assessment of IR in postmenopausal women is of great significance for predicting their long-term prognostic risks. The eGDR, originally designed to assess the severity of IR in individuals with type 1 diabetes by incorporating WC, HbA1c, and hypertension status (32). However, the utility of eGDR in predicting adverse outcomes extends beyond its original application in type 1 diabetes. It has been shown in various studies to be associated with impaired fasting glucose, impaired glucose tolerance, and adverse cardiovascular outcomes in both non-diabetic individuals and those with type 2 diabetes (16, 20, 21, 33). According to Zhang et al., lower eGDR levels were associated with an elevated likelihood of incident CVDs among non-diabetic participants (16). Zabala et al. identified a significant association between reduced eGDR and an elevated risk of stroke and post-stroke mortality in individuals with type 2 diabetes (34). Similarly, Nyström et al. demonstrated that a low eGDR, regardless of other cardiovascular and metabolic factors, was linked to a higher risk of long-term all-cause mortality in type 2 diabetes patients undergoing coronary artery bypass surgery (35). Chen et al. found that eGDR is strongly linked to metabolic syndrome prevalence and better predicts all-cause and cardiovascular mortality compared to other IR indices (36). In postmenopausal women, where the interplay between hormonal changes, visceral fat accumulation, and IR is complex, eGDR could serve as a valuable tool for early identification of individuals at high risk of long-term mortality. The eGDR might capture estrogen-mediated metabolic changes more effectively than traditional IR markers because its components directly reflect the postmenopausal metabolic phenotype: WC captures the shift toward visceral adiposity, HbA1c reflects long-term glycemic dysregulation, and hypertension status indicates metabolic syndrome-related vascular dysfunction. Our results demonstrated that eGDR provided superior discriminative ability for both all-cause and cardiovascular mortality prediction compared to these alternative IR markers, with significant improvements in C-statistics, NRI, and IDI. While direct validation against gold-standard measures such as the hyperinsulinemic-euglycemic clamp would be ideal, these are not available in NHANES. Although our subgroup analyses did not reveal significant statistical interactions across racial/ethnic groups, the observed variations in eGDR associations may reflect genetic differences in insulin sensitivity and estrogen metabolism pathways, socioeconomic disparities in healthcare access, and varying baseline comorbidity burden across populations. These observations warrant further investigation in larger, ethnically diverse cohorts to better understand potential population-specific effects.

Our findings reveal a potential threshold for eGDR’s effect on overall survival, which may reflect the complex effect of insulin sensitivity on multiple causes of death. Beyond a certain threshold, further increases in eGDR do not necessarily enhance survival, suggesting a saturation effect. Prior studies by Guo et al. also reported the existence of this nonlinear relationship in the general population (37). Additionally, this observation might align with the U-shaped relationship between WC, HbA1c, and all-cause mortality (38). Elevated WC suggests central obesity, whereas a low WC might indicate malnutrition or diminished muscle mass, both of which are linked to increased all-cause mortality (39, 40). Similarly, excessively low HbA1c levels may reflect overly stringent glycemic control, heightening the risk of hypoglycemia, while elevated HbA1c levels are associated with poor glycemic management and worse prognostic outcomes (41, 42). Unlike all-cause mortality, a reduction in eGDR shows a more direct and linear association with cardiovascular mortality. As an index used to estimate insulin sensitivity based on metabolic parameters, eGDR is closely associated with the primary direct drivers of cardiovascular mortality, namely IR and metabolic dysfunction. Liao et al.’s study focused on patients with diabetes and prediabetes, also revealing a comparable negative linear correlation between eGDR and cardiovascular diseases (43). Song et al. also observed a linear pattern between eGDR and cardiovascular mortality in those with nonalcoholic fatty liver disease (NAFLD) (44). Further subgroup analysis revealed that the influence of eGDR on survival is affected by age. The relationship between eGDR and mortality is more significant in younger individuals than in older populations. Younger individuals generally have higher metabolic reserves and stronger compensatory mechanisms, making a decline in eGDR (indicating worsening IR) a more critical health indicator (37). In older individuals, the cumulative effects of chronic diseases and other risk factors may obscure the role of eGDR (37).

Several important limitations warrant consideration. Firstly, the self-reported menopause history questionnaire, which lacks measurements of hormone levels and associated symptoms, might poses certain limitations to the reliability of the results. Self-reported age at last menstrual period might also be subject to recall bias, particularly among older participants. Additionally, due to differences in questionnaire design across NHANES cycles regarding menstrual irregularity, women with hysterectomy were included, some of whom may have retained their ovaries (**Supplementary Materials: Supplementary Table S6**). Secondly, the retrospective design limits causal inference. while we observed strong associations between eGDR and mortality, we cannot establish causality. Thirdly, although it relies on a large-scale population cohort, the collection of anthropometric data and laboratory indicators was restricted to baseline. Future prospective studies measuring eGDR trajectory changes and incident cardiovascular outcomes would provide stronger evidence. Finally, generalizability to non-Western populations remains uncertain, as NHANES data may not adequately represent women in low- to middle-income countries where lifestyle factors, healthcare access, and genetic backgrounds differ substantially.

## 5 Conclusion

Elevated eGDR levels are significantly associated with reduced risks of all-cause and cardiovascular mortality in postmenopausal women, suggesting that eGDR might serve as a potential predictive marker for mortality risk assessment in this population.

## Data Availability

Publicly available datasets were analyzed in this study. This data can be found here: https://wwwn.cdc.gov/nchs/nhanes.
