# Supplementary material for: Insulin resistance assessed by estimated glucose disposal rate is associated with all-cause and cardiovascular mortality among postmenopausal women
Source: Front Endocrinol (Lausanne). 2025 Jul 30;16:1583991. doi: 10.3389/fendo.2025.1583991 (PMC12343283; doi:10.3389/fendo.2025.1583991)
Supplement: Supplementary file 1 [file DataSheet1.docx]

**Insulin resistance assessed by estimated glucose disposal rate is associated with all-cause and cardiovascular mortality among postmenopausal women**

**Supplementary Materials**

**Table S1.** The variance inflation factors for covariates in models.

**Table S2.** Threshold effect analysis of eGDR on all-cause mortality.

**Table S3.** Relationship between eGDR and mortality after excluding individuals who died within the first two years.

**Table S4.** Relationship between eGDR and mortality after excluding participants with baseline diabetes.

**Table S5.** Relationship between eGDR and mortality after excluding participants with incomplete covariate data.

**Table S6.** NHANES Reproductive Questionnaire (Reasons irregular menstruation).

| **Table S1**. The variance inflation factors (VIFs) for covariates in models. | |
| --- | --- |
| Variable | VIF |
| Age | 1.6 |
| Age at menopause | 1 |
| Race | 1.1 |
| Marital status | 1.1 |
| Education level | 1.3 |
| PIR | 1.3 |
| Smoking history | 1.1 |
| Alcohol consumption | 1.1 |
| BMI | 1.7 |
| Albumin | 1.1 |
| ALT | 2.8 |
| AST | 2.7 |
| TG | 1.4 |
| HDL | 1.5 |
| LDL | 1.1 |
| SUA | 1.4 |
| eGFR | 1.7 |
| SUA | 1.4 |
| Diabetes | 1.3 |
| eGDR | 2 |
| Abbreviation: PIR: family income-to-poverty ratio, BMI: body mass index, ALT: aspartate aminotransferase, AST: alanine aminotransferase, TG: triglyceride, HDL-c: high-density lipoprotein cholesterol, LDL-c: low-density lipoprotein cholesterol, SUA: serum uric acid, eGFR: estimated glomerular filtration rate, eGDR: estimated glucose disposal rate. | |

| **Table S2.** Threshold effect analysis of eGDR on all-cause mortality. | |
| --- | --- |
| Model | HR (95% CI) P value |
| Total | 0.960 (0.936, 0.984) 0.001 |
| Breakpoint (K) | 5.11 |
| HR1 (<5.11) | 0.884 (0.830, 0.941) <0.001 |
| HR2 (>5.11) | 0.980 (0.952, 1.009) 0.170 |
| HR2/HR1 | 1.109 (1.031, 1.193) 0.005 |
| P for logarithmic likelihood ratio | 0.006 |
| adjusted for age, race, marital status, education level, PIR, smoking history, alcohol consumption, age at menopause, BMI, diabetes, albumin, AST, ALT, TG, HDL-c, LDL-c, SUA, and eGFR.  Abbreviation: HR: hazards ratio, 95% CI: 95% confidence interval. | |

**Table S3.** Relationship between eGDR and mortality after excluding individuals who died within the first two years.

|  | **Q1** |  | **Q2** | |  | **Q3** | |  | **Q4** | | ***P* for**  **trend** |  |
| --- | --- | --- | --- | --- | --- | --- | --- | --- | --- | --- | --- | --- |
|  | HR (95% CI) |  | HR (95% CI) | *P* value |  | HR (95% CI) | *P* value |  | HR (95% CI) | *P* value |  |  |
| **All-cause mortality** | |  |  |  |  |  |  |  |  |  |  |  |
| **No. of cases/N** | | 506/2287 |  | 517/2284 |  |  | 528/2288 |  |  | 375/2287 |  |  |
| **Model 1** | | Ref. |  | 0.942 (0.834, 1.065) | 0.343 |  | 0.929 (0.822, 1.049) | 0.235 |  | 0.586 (0.513, 0.670) | <0.001 | <0.001 |
| **Model 2** | | Ref. |  | 0.713 (0.630, 0.807) | <0.001 |  | 0.777 (0.687, 0.879) | <0.001 |  | 0.637 (0.557, 0.730) | <0.001 | <0.001 |
| **Model 3** | | Ref. |  | 0.721 (0.637, 0.817) | <0.001 |  | 0.783 (0.691, 0.886) | <0.001 |  | 0.654 (0.570, 0.750) | <0.001 | <0.001 |
| **Model 4** | | Ref. |  | 0.785 (0.680, 0.906) | <0.001 |  | 0.837 (0.711, 0.985) | 0.032 |  | 0.721 (0.603, 0.863) | <0.001 | 0.003 |
| **Cardiovascular mortality** | |  |  |  |  |  |  |  |  |  |  |  |
| **No. of cases/N** | | 173/2287 |  | 178/2284 |  |  | 155/2288 |  |  | 101/2287 |  |  |
| **Model 1** | | Ref. |  | 0.948 (0.769, 1.168) | 0.614 |  | 0.797 (0.641, 0.990) | 0.040 |  | 0.461 (0.361, 0.590) | <0.001 | <0.001 |
| **Model 2** | | Ref. |  | 0.661 (0.534, 0.817) | <0.001 |  | 0.624 (0.501, 0.778) | <0.001 |  | 0.495 (0.386, 0.635) | <0.001 | <0.001 |
| **Model 3** | | Ref. |  | 0.675 (0.545, 0.835) | <0.001 |  | 0.639 (0.512, 0.798) | <0.001 |  | 0.514 (0.399, 0.661) | <0.001 | <0.001 |
| **Model 4** | | Ref. |  | 0.805 (0.630, 1.029) | 0.084 |  | 0.737 (0.550, 0.987) | 0.041 |  | 0.635 (0.459, 0.879) | 0.006 | 0.008 |

Estimates are hazard ratios (95%CI) from Cox proportional hazard models.

Model 1: no adjusted. Model 2: adjusted for age and race. Model 3: adjusted for age, race, marital status, education level, PIR, smoking history, and alcohol consumption. Model 4: adjusted for age, race, marital status, education level, PIR, smoking history, alcohol consumption, age at menopause, BMI, diabetes, albumin, AST, ALT, TG, HDL-c, LDL-c, SUA, and eGFR.

Abbreviations: HR: hazard ratio, 95%CI: 95% confidence interval.

**Table S4.** Relationship between eGDR and mortality after excluding participants with baseline diabetes.

|  | **Q1** |  | **Q2** | |  | **Q3** | |  | **Q4** | | ***P* for**  **trend** |  |
| --- | --- | --- | --- | --- | --- | --- | --- | --- | --- | --- | --- | --- |
|  | HR (95% CI) |  | HR (95% CI) | *P* value |  | HR (95% CI) | *P* value |  | HR (95% CI) | *P* value |  |  |
| **All-cause mortality** | |  |  |  |  |  |  |  |  |  |  |  |
| **No. of cases/N** | | 358/1765 |  | 446/1762 |  |  | 395/1766 |  |  | 302/1765 |  |  |
| **Model 1** | | Ref. |  | 1.231 (1.071, 1.415) | 0.003 |  | 1.065 (0.923, 1.228) | 0.391 |  | 0.736 (0.632, 0.858) | <0.001 | <0.001 |
| **Model 2** | | Ref. |  | 0.981 (0.853, 1.129) | 0.788 |  | 1.066 (0.924, 1.231) | 0.382 |  | 0.900 (0.771, 1.050) | 0.180 | 0.417 |
| **Model 3** | | Ref. |  | 0.977 (0.849, 1.125) | 0.749 |  | 1.046 (0.905, 1.209) | 0.542 |  | 0.912 (0.780, 1.067) | 0.249 | 0.473 |
| **Model 4** | | Ref. |  | 0.893 (0.758, 1.051) | 0.174 |  | 0.936 (0.788, 1.113) | 0.455 |  | 0.803 (0.660, 0.977) | 0.029 | 0.071 |
| **Cardiovascular mortality** | |  |  |  |  |  |  |  |  |  |  |  |
| **No. of cases/N** | | 131/1765 |  | 141/1762 |  |  | 112/1766 |  |  | 77/1765 |  |  |
| **Model 1** | | Ref. |  | 1.061 (0.836, 1.345) | 0.628 |  | 0.823 (0.639, 1.059) | 0.130 |  | 0.512 (0.386, 0.679) | <0.001 | <0.001 |
| **Model 2** | | Ref. |  | 0.798 (0.628, 1.015) | 0.066 |  | 0.790 (0.613, 1.018) | 0.069 |  | 0.635 (0.478, 0.843) | 0.002 | 0.003 |
| **Model 3** | | Ref. |  | 0.813 (0.638, 1.035) | 0.093 |  | 0.793 (0.614, 1.025) | 0.077 |  | 0.650 (0.488, 0.867) | 0.003 | 0.004 |
| **Model 4** | | Ref. |  | 0.754 (0.567, 1.002) | 0.052 |  | 0.688 (0.504, 0.940) | 0.019 |  | 0.575 (0.401, 0.825) | 0.003 | 0.003 |

Estimates are hazard ratios (95%CI) from Cox proportional hazard models.

Model 1: no adjusted. Model 2: adjusted for age and race. Model 3: adjusted for age, race, marital status, education level, PIR, smoking history, and alcohol consumption. Model 4: adjusted for age, race, marital status, education level, PIR, smoking history, alcohol consumption, age at menopause, BMI, albumin, AST, ALT, TG, HDL-c, LDL-c, SUA, and eGFR.

Abbreviations: HR: hazard ratio, 95%CI: 95% confidence interval.

**Table S5.** Relationship between eGDR and mortality after excluding participants with incomplete covariate data.

|  | **Q1** |  | **Q2** | |  | **Q3** | |  | **Q4** | | ***P* for**  **trend** |  |
| --- | --- | --- | --- | --- | --- | --- | --- | --- | --- | --- | --- | --- |
|  | HR (95% CI) |  | HR (95% CI) | *P* value |  | HR (95% CI) | *P* value |  | HR (95% CI) | *P* value |  |  |
| **All-cause mortality** | |  |  |  |  |  |  |  |  |  |  |  |
| **No. of cases/N** | | 216/980 |  | 242/979 |  |  | 231/980 |  |  | 160/980 |  |  |
| **Model 1** | | Ref. |  | 1.030 (0.857, 1.237) | 0.753 |  | 0.986 (0.819, 1.188) | 0.884 |  | 0.607 (0.494, 0.745) | <0.001 | <0.001 |
| **Model 2** | | Ref. |  | 0.785 (0.651, 0.945) | 0.011 |  | 0.864 (0.716, 1.042) | 0.127 |  | 0.659 (0.535, 0.811) | <0.001 | <0.001 |
| **Model 3** | | Ref. |  | 0.818 (0.678, 0.986) | 0.035 |  | 0.896 (0.741, 1.082) | 0.253 |  | 0.723 (0.585, 0.893) | 0.003 | 0.013 |
| **Model 4** | | Ref. |  | 0.812 (0.657, 1.004) | 0.054 |  | 0.907 (0.708, 1.161) | 0.437 |  | 0.747 (0.569, 0.981) | 0.036 | 0.112 |
| **Cardiovascular mortality** | |  |  |  |  |  |  |  |  |  |  |  |
| **No. of cases/N** | | 77/980 |  | 88/979 |  |  | 69/980 |  |  | 35/980 |  |  |
| **Model 1** | | Ref. |  | 1.051 (0.774, 1.427) | 0.751 |  | 0.828 (0.598, 1.146) | 0.254 |  | 0.372 (0.250, 0.555) | <0.001 | <0.001 |
| **Model 2** | | Ref. |  | 0.728 (0.533, 0.995) | 0.047 |  | 0.678 (0.488, 0.943) | 0.021 |  | 0.399 (0.266, 0.599) | <0.001 | <0.001 |
| **Model 3** | | Ref. |  | 0.764 (0.557, 1.048) | 0.095 |  | 0.714 (0.511, 0.997) | 0.048 |  | 0.436 (0.288, 0.661) | <0.001 | <0.001 |
| **Model 4** | | Ref. |  | 0.828 (0.578, 1.186) | 0.303 |  | 0.818 (0.528, 1.266) | 0.367 |  | 0.513 (0.308, 0.855) | 0.010 | 0.016 |

Estimates are hazard ratios (95%CI) from Cox proportional hazard models.

Model 1: no adjusted. Model 2: adjusted for age and race. Model 3: adjusted for age, race, marital status, education level, PIR, smoking history, and alcohol consumption. Model 4: adjusted for age, race, marital status, education level, PIR, smoking history, alcohol consumption, age at menopause, BMI, diabetes, albumin, AST, ALT, TG, HDL-c, LDL-c, SUA, and eGFR.

Abbreviations: HR: hazard ratio, 95%CI: 95% confidence interval.

**Table S6.** NHANES Reproductive Questionnaire (Reasons irregular menstruation).

NHANES 1990-2000, 2001-2002

What is the reason that {you have/SP has} not had regular periods in the past 12 months?

| Code or Value | Value Description |
| --- | --- |
| 1 | Pregnant now |
| 2 | Breast feeding |
| 3 | Pregnant in past year |
| 4 | Periods usually irregular |
| 5 | **Going-gone through menopause** |
| 6 | Medical conditions-treatments |
| 77 | Refused |
| 99 | Don't know |
| . | Missing |

NHANES 2003-2004, 2005-2006, 2007-2008, 2009-2010, 2011-2012,

What is the reason that {you have/SP has} not had regular periods in the past 12 months?

| Code or Value | Value Description |
| --- | --- |
| 1 | Pregnancy |
| 2 | Breast feeding |
| 7 | **Menopause/Hysterectomy** |
| 8 | Medical conditions/treatments |
| 9 | Other |
| 77 | Refused |
| 99 | Don't know |
| . | Missing |

NHANES 2013-2014, 2015-2016, 2017-2018

What is the reason that {you have/SP has} not had regular periods in the past 12 months?

| Code or Value | Value Description |
| --- | --- |
| 1 | Pregnancy |
| 2 | Breast feeding |
| 3 | **Hysterectomy** |
| 7 | **Menopause/Change of life** |
| 9 | Other |
| 77 | Refused |
| 99 | Don't know |
| . | Missing |
